# Supplementary material for: Crystallization of Form II Paracetamol with the Assistance of Carboxylic Acids toward Batch and Continuous Processes
Source: Pharmaceutics. 2022 May 20;14(5):1099. doi: 10.3390/pharmaceutics14051099 (PMC9147162; doi:10.3390/pharmaceutics14051099)
Supplement: Supplementary file 1 [file pharmaceutics-14-01099-s001.zip › pharmaceutics-1696387-supplementary.pdf]

Supplementary Materials

Crystallization of Form II Paracetamol with the Assistance of Carboxylic Acids toward Batch and Continuous Processes

Kuan Lin Yeh, Hung Lin Lee, Tu Lee*

Department of Chemical and Materials Engineering, National Central University

300 Zhongda Road, Zhongli District, Taoyuan City 320317, Taiwan R.O.C.

This PDF file includes:

* Corresponding Author. Tel: +886-3-4227151 ext. 34204. Fax: +886-3-4252296.

Email: tulee@cc.ncu.edu.tw

Table S1. Experimental conditions for batch cooling crystallization using a 0.5 L glass vessel (Figure S1) in Expt. 1 to 20.

Expt.	PCA (g)	FUM (g)	OXA (g)	Water (mL)	Agitation speed (rpm)	Residence time (h)
1	20	-	-	340	300	1.5
2	20	-	-	340	200	1.5
3	20	-	-	340	100	1.5
4	20	4	-	340	300	1.5
5	20	4	-	340	200	1.5
6	20	4	-	340	100	1.5
7	20	10	-	340	300	1.5
8	20	10	-	340	200	1.5
9	20	10	-	340	100	1.5
10	20	-	12	340	300	1.5
11	20	-	12	340	200	1.5
12	20	-	12	340	100	1.5
13	20	-	24	340	300	1.5
14	20	-	24	340	200	1.5
15	20	-	24	340	100	1.5
16	20	-	-	340	0	4
17	20	4	-	340	0	4
18	20	10	-	340	0	4
19	20	-	12	340	0	4
20	20	-	24	340	0	4

Table S2. Experimental conditions for continuous cooling crystallization using a tubular crystallizer (Figure S2) in Expt. 21 to 28.

Expt.	PCA (g)	FUM (g)	Water (mL)	Flow rate (mL/min)
21	20	4	340	75
22	20	4	340	150
23	20	6	340	75
24	20	6	340	150
25	20	10	340	75
26	20	10	340	150
27	15	7.5	340	75
28	15	7.5	340	150

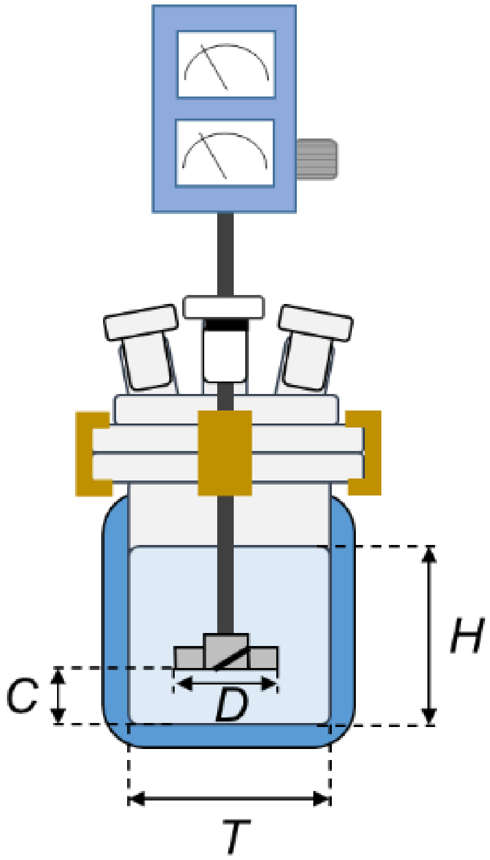

Figure S1. Configuration of a 0.5 L jacketed glass vessel as a batch crystallizer: T = 78 mm, H = 71 mm, D = 30 mm, and C = 23 mm.

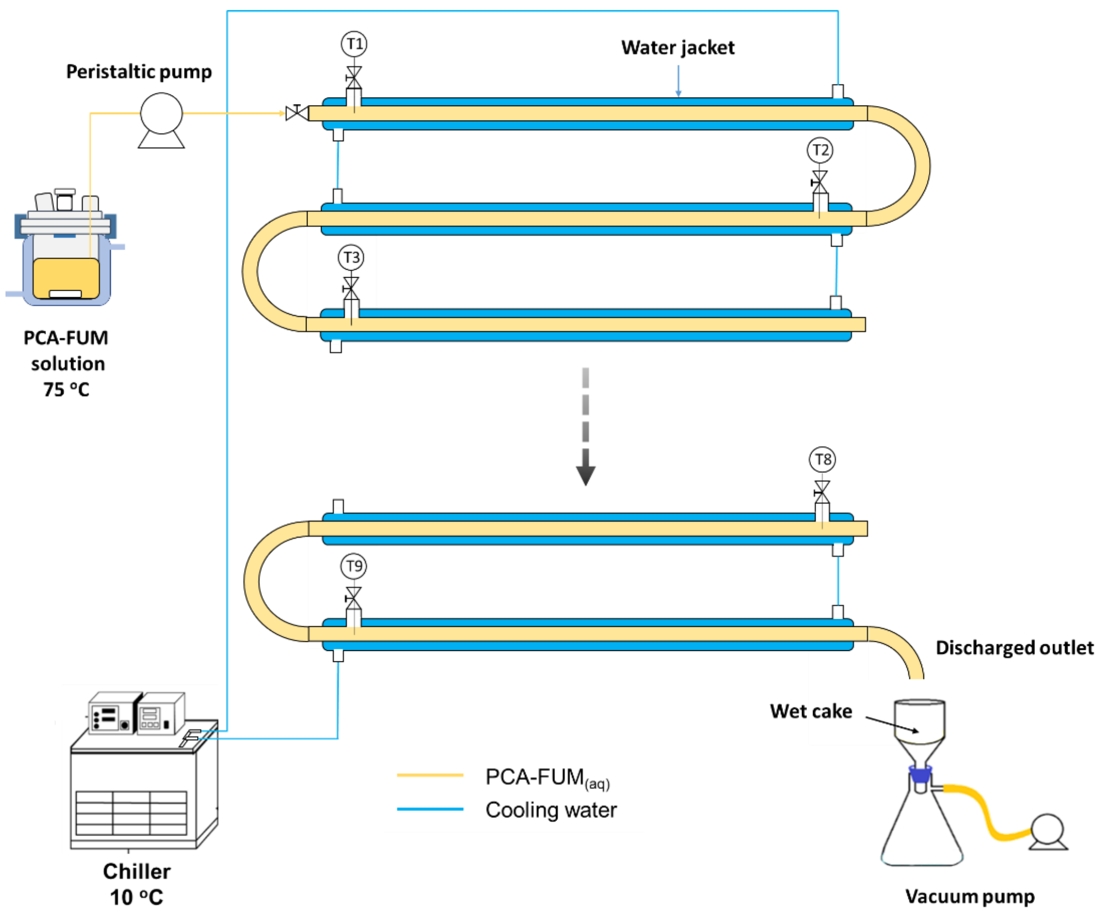

Figure S2. Experimental setup for continuous crystallization using a tubular crystallizer. Nine thermocouples labeled as T1 to T9 were inserted to record a temperature profile thoroughly in the tubular crystallizer.

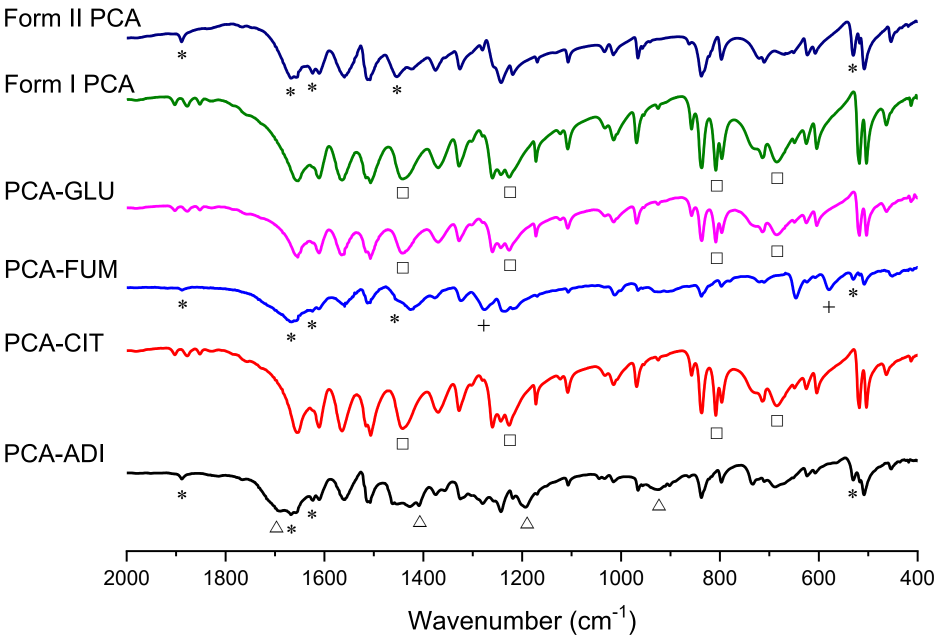
Form II PCA
Form I PCA
PCA-GLU
PCA-FUM
PCA-CIT
PCA-ADI
2000
1800
1600
1400
1200
1000
800
600
400
Wavenumber (cm^{-1})

(a)

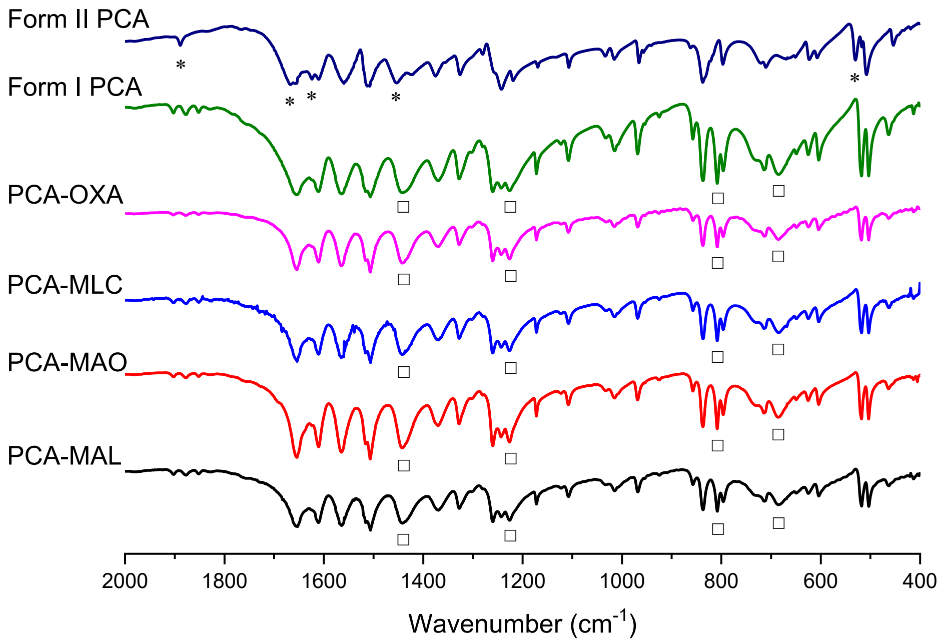
Form II PCA
Form I PCA
PCA-OXA
PCA-MLC
PCA-MAO
PCA-MAL
2000
1800
1600
1400
1200
1000
800
600
400
Wavenumber (cm^{-1})

(b)

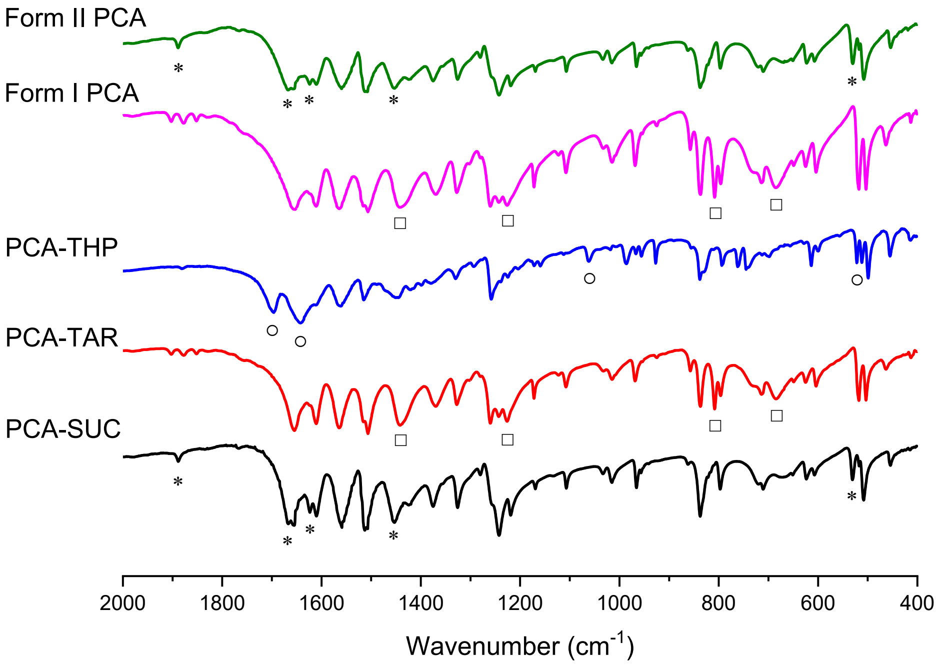

(c)

Figure S3. FTIR spectra of the PCA crystals produced by Screening Method 1 with different additives. The characteristic peaks of Form I PCA, Form II PCA, ADI, FUM, and 1:1 PCA-THP co-crystal are labeled by □, ∗, △, +, and ○, respectively.

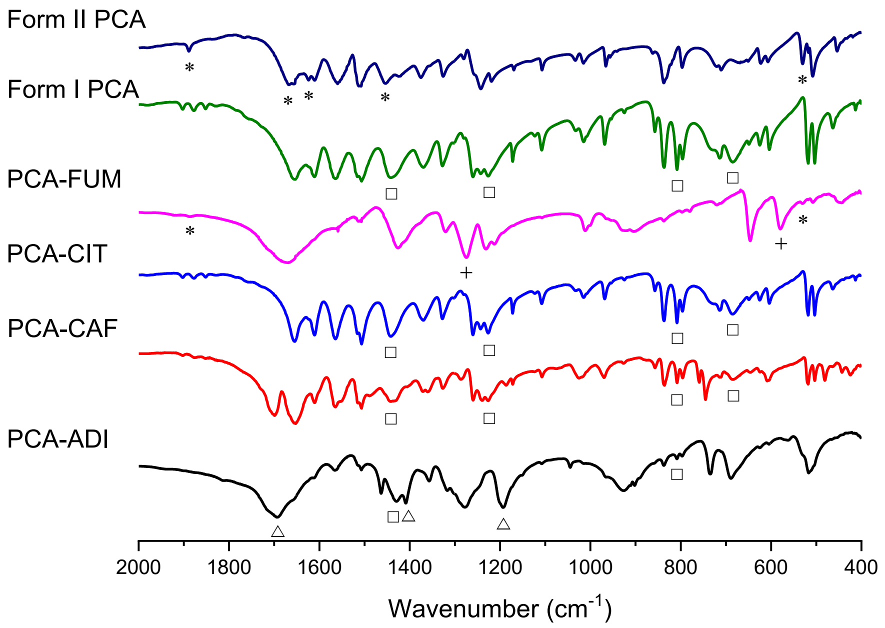
Form II PCA
Form I PCA
PCA-FUM
PCA-CIT
PCA-CAF
PCA-ADI
2000
1800
1600
1400
1200
1000
800
600
400
Wavenumber (cm-1)

(a)

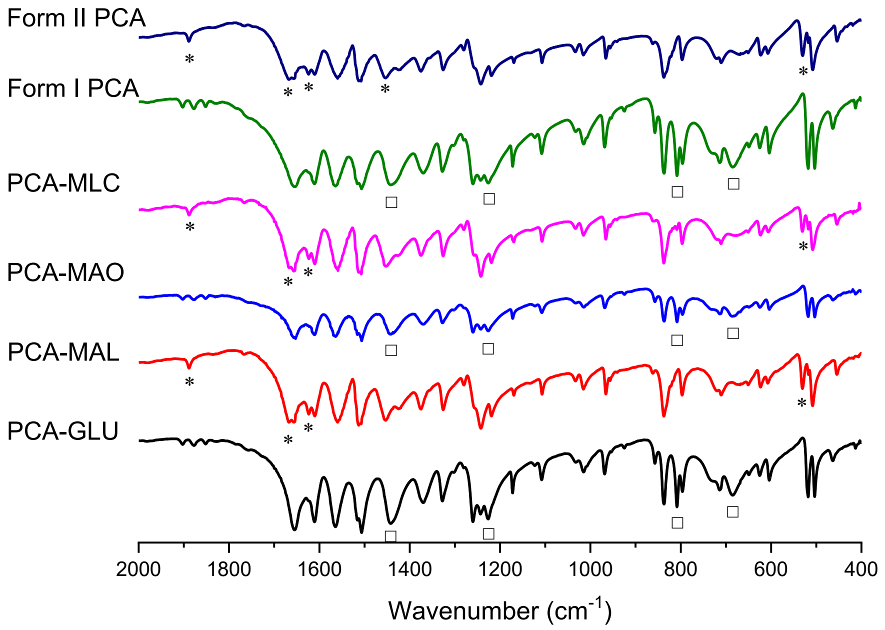
Form II PCA
Form I PCA
PCA-MLC
PCA-MAO
PCA-MAL
PCA-GLU
2000
1800
1600
1400
1200
1000
800
600
400
Wavenumber (cm-1)

(b)

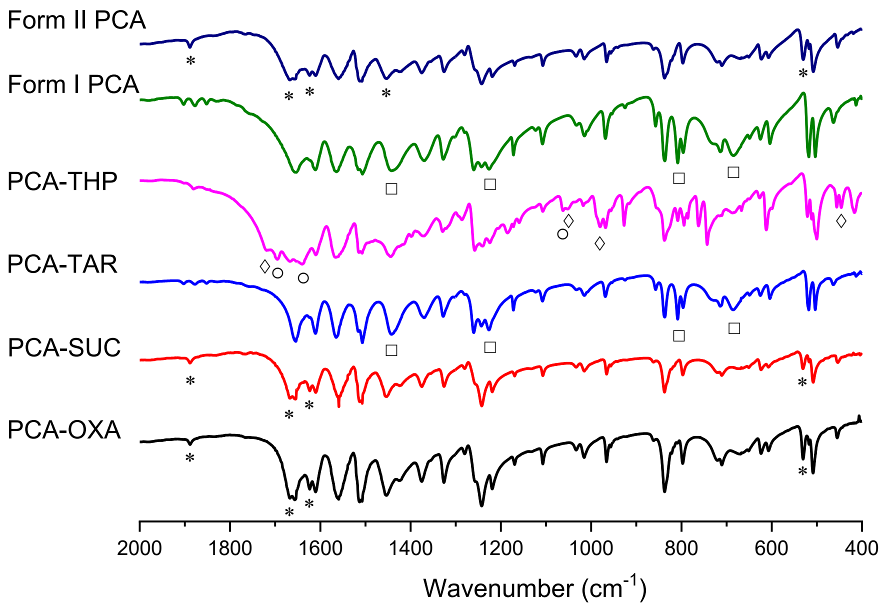

(c)

Figure S4. FTIR spectra of the PCA crystals produced by Screening Method 2 with different additives. The characteristic peaks of Form I PCA, Form II PCA, ADI, FUM, THP, and 1:1 PCA-THP co-crystal are labeled by □, ∗, △, +, ◊, and ○, respectively.

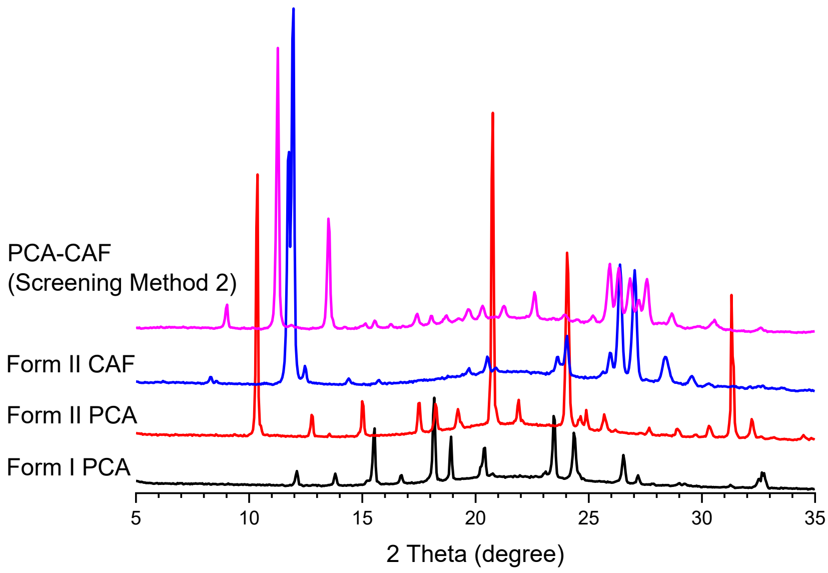

Figure S5. PXRD patterns of Forms I and II PCA, Form II CAF, and PCA-CAF co-crystal (Screening Method 2).

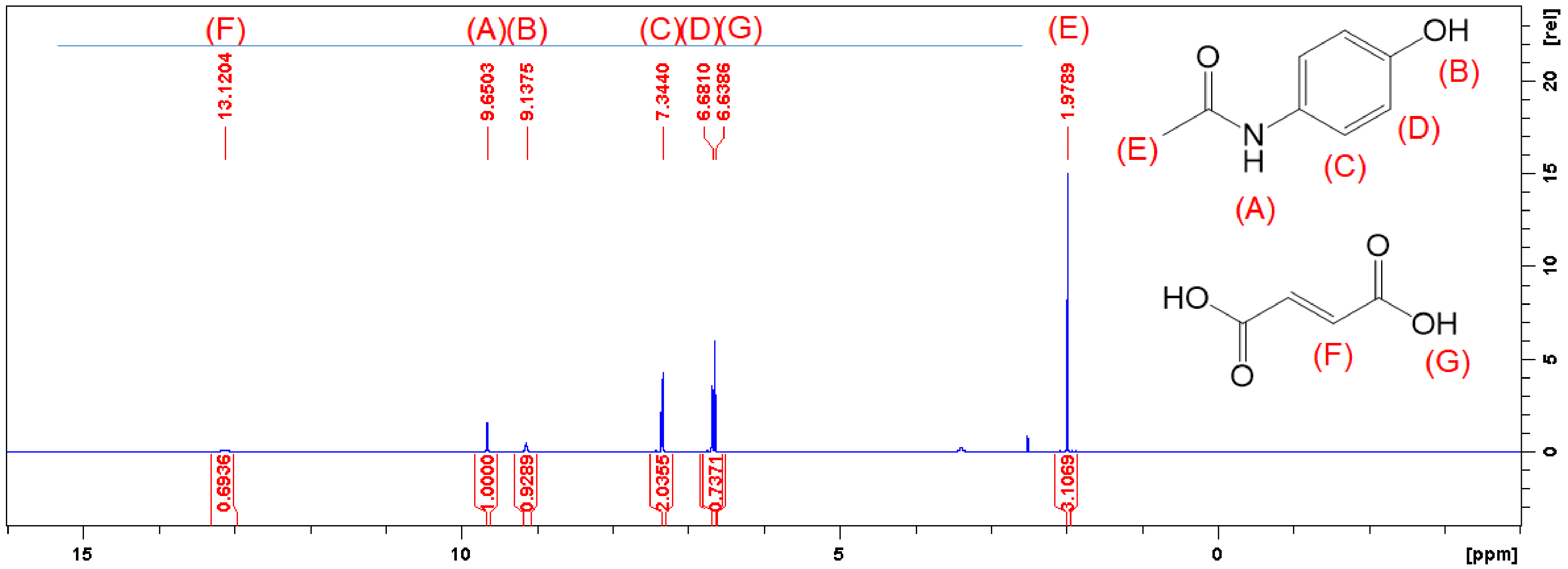

Figure S6. ^{1}H NMR spectrum of Form II PCA produced from the PCA-MAL solution by Screening Method 2.

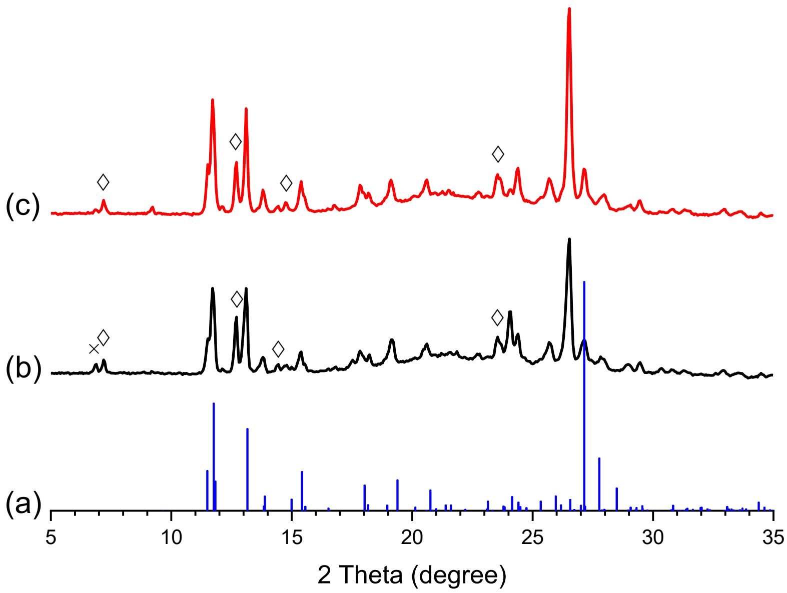

Figure S7. (a) Theoretical diffraction pattern of 1:1 PCA-THP co-crystal from the Cambridge Crystallographic Data Centre (CCDC) with a CCDC identifier: KIGLUI, and PXRD patterns of the PCA crystals produced by (b) Screening Method 1 and (c) Screening Method 2 in the presence of THP. The characteristic peaks of Form II THP and unknown species are labeled by ◊ and ×, respectively.

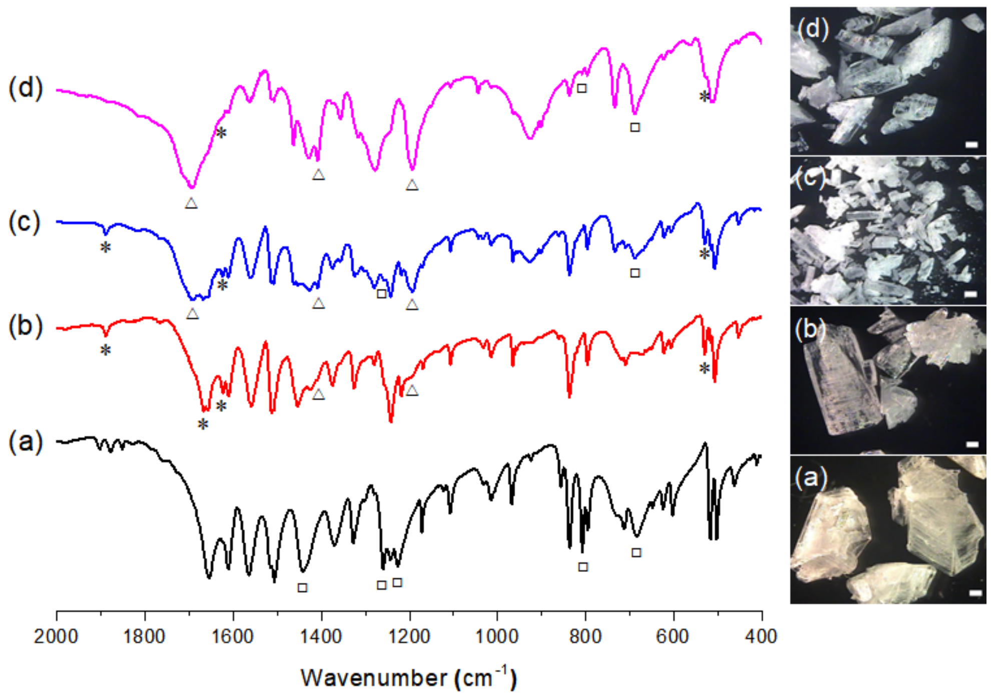

Figure S8. FTIR spectra and OM images of the PCA crystals produced by cooling crystallization at (a) 25, (b) 50, (c) 75, and (d) 100 wt% of ADI (scale bar = 500 μm). The characteristic peaks of Form I PCA, Form II PCA, and ADI are labeled by □, ∗, and △, respectively.

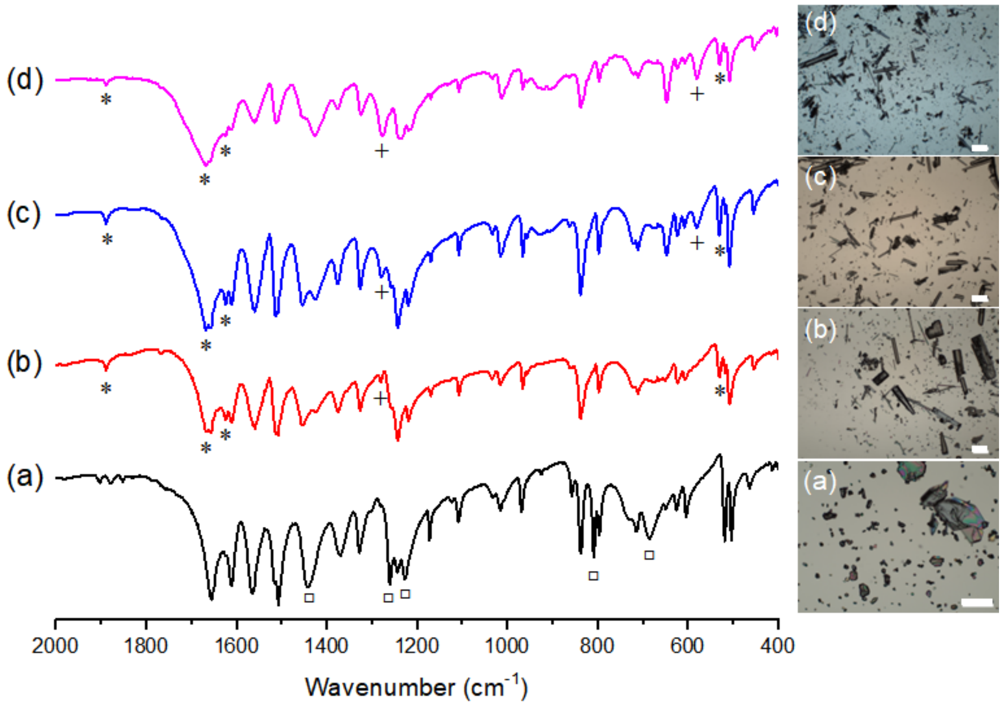

Figure S9. FTIR spectra and OM images of the PCA crystals produced by cooling crystallization at (a) 10, (b) 20, (c) 30, and (d) 50 wt% of FUM (scale bar = 200 μm). The characteristic peaks of Form I PCA, Form II PCA, and FUM are labeled by □, ∗, and +, respectively.

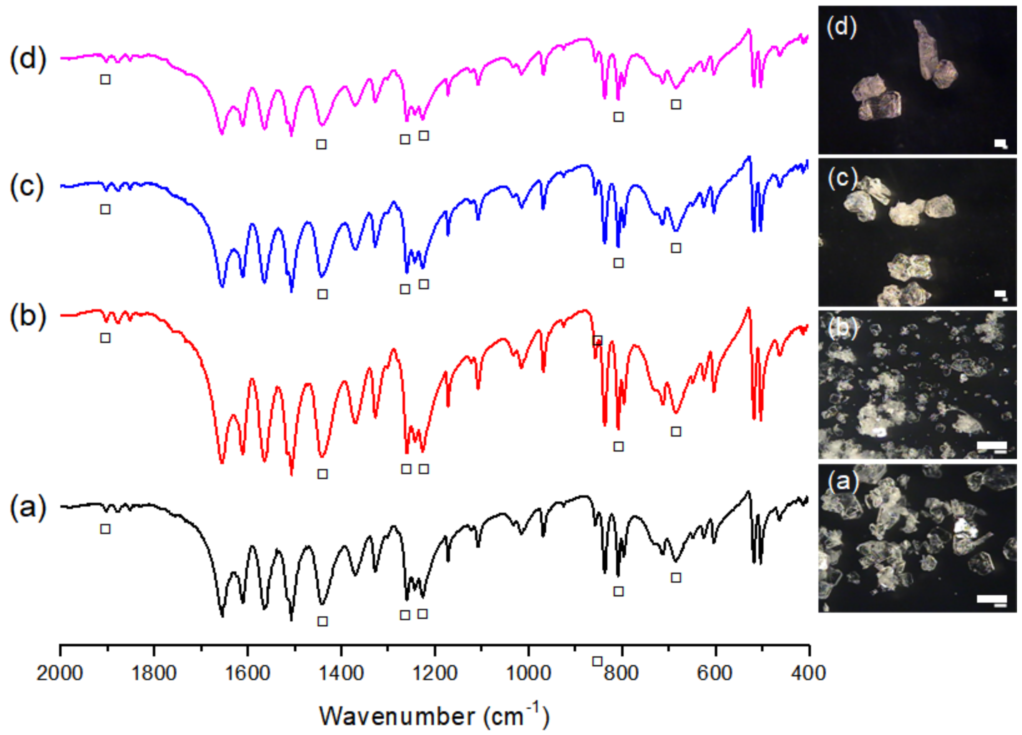

Figure S10. FTIR spectra and OM images of the PCA crystals produced by cooling crystallization at (a) 25, (b) 50, (c) 75, and (d) 100 wt% of MLC (scale bar = 500 μm). The characteristic peaks of Form I PCA are labeled by □.

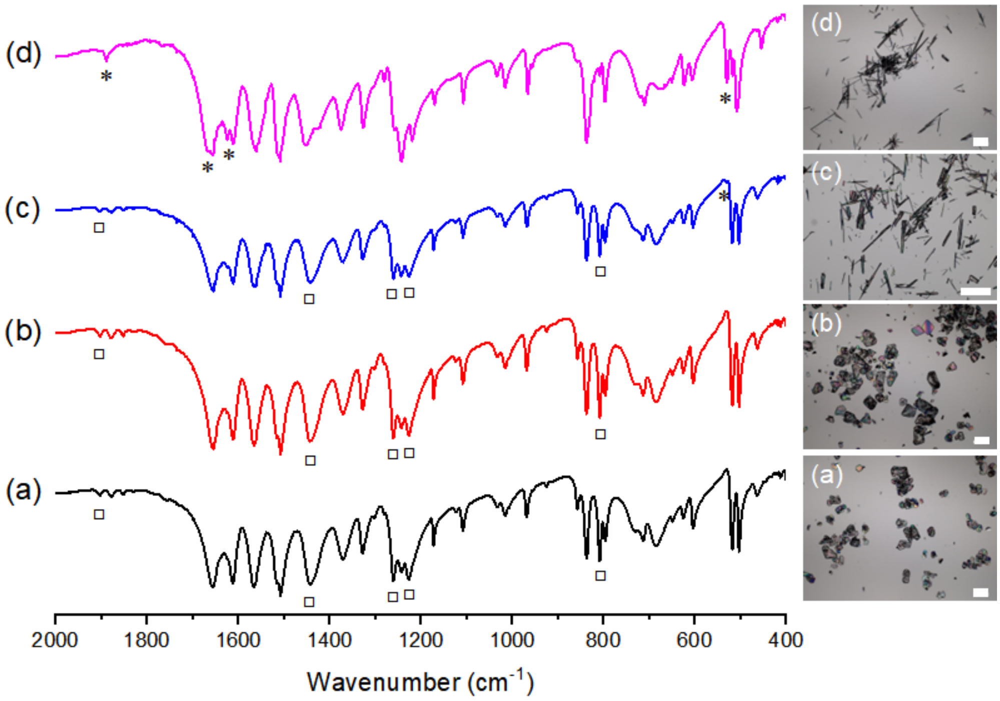

Figure S11. FTIR spectra and OM images of the PCA crystals produced by cooling crystallization at (a) 30, (b) 60, (c) 90, and (d) 120 wt% of OXA (scale bar = 200 μm). The characteristic peaks of Form I PCA and Form II PCA are labeled by □ and ∗, respectively.

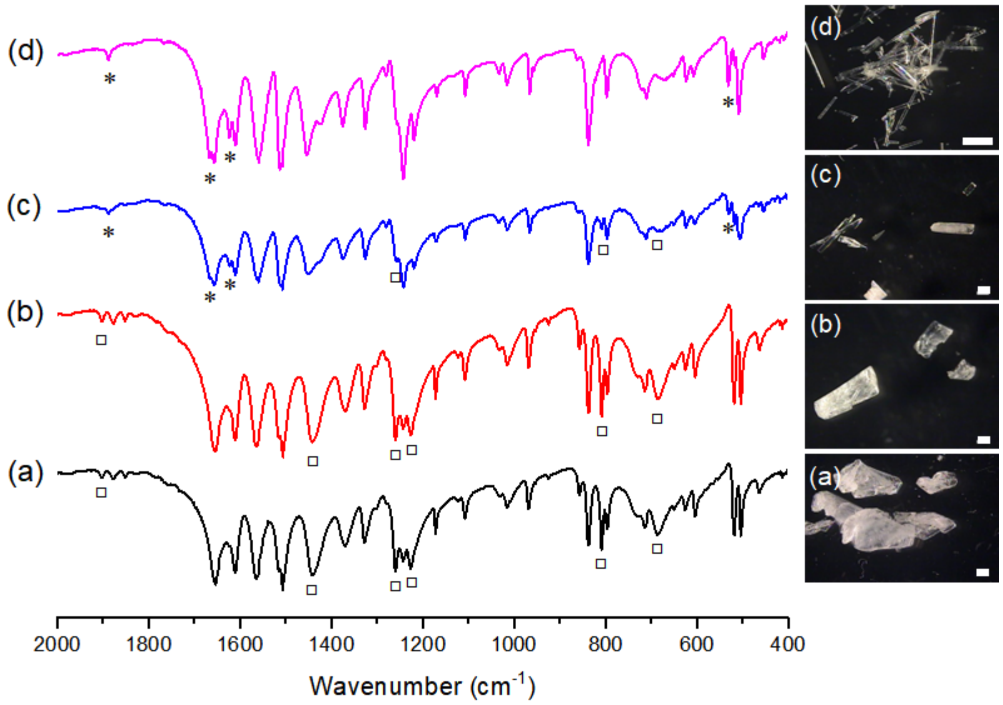

Figure S12. FTIR spectra and OM images of the PCA crystals produced by cooling crystallization at (a) 25, (b) 50, (c) 75, and (d) 100 wt% of SUC (scale bar = 500 μm). The characteristic peaks of Form I PCA and Form II PCA are labeled by □ and ∗, respectively.

Expt.	At induction temp.	5 min after induction	At the end
1	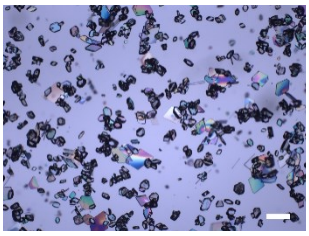	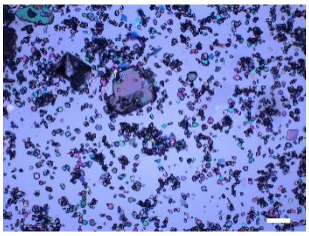	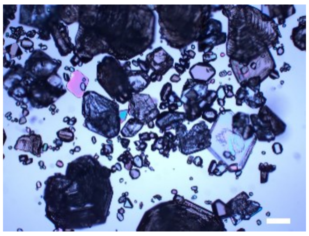
2	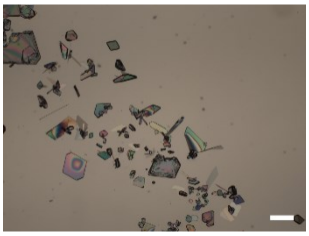	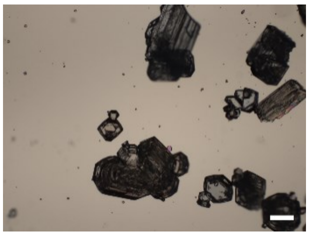	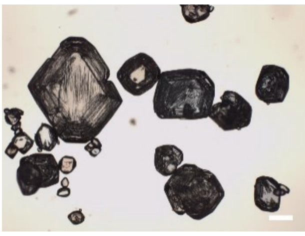
3	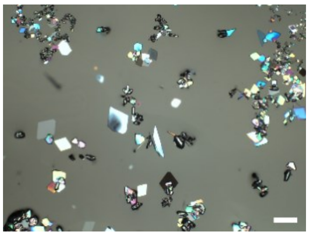	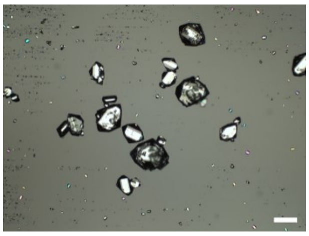	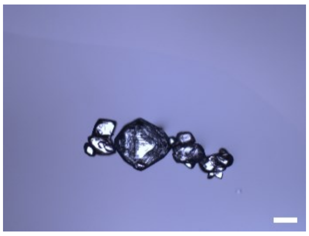

Figure S13. OM images of the PCA crystals produced by batch cooling crystallization using a 0.5 L-sized glass vessel in Expt. 1 to 3 (scale bar = 200 μm).

Expt.	At induction temp.	5 min after induction	At the end
4	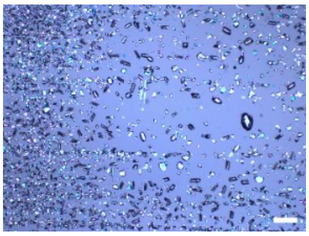	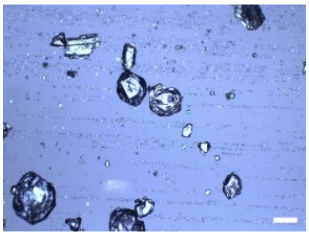	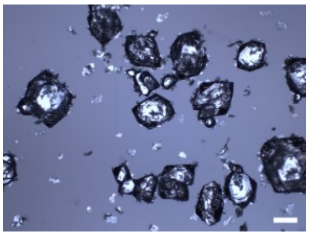
5	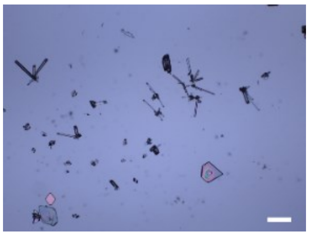	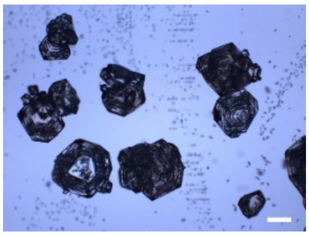	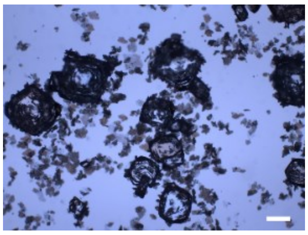
6	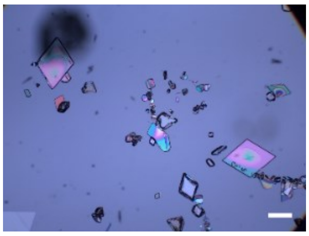	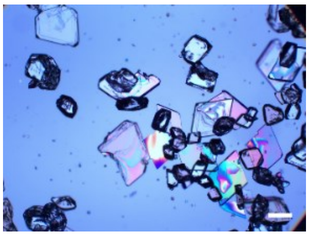	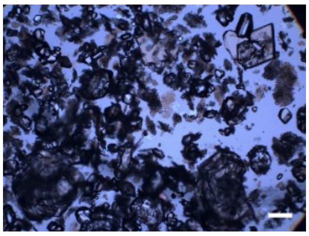
7	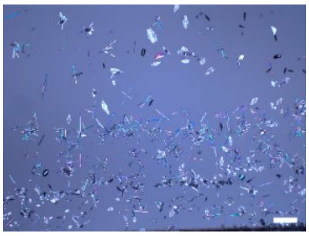	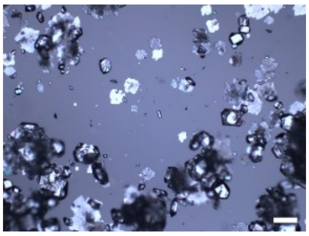	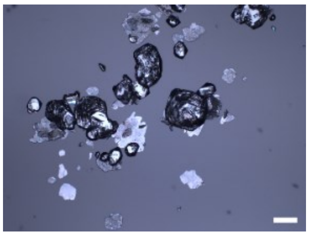
8	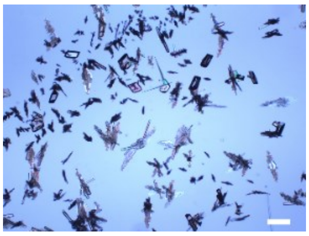	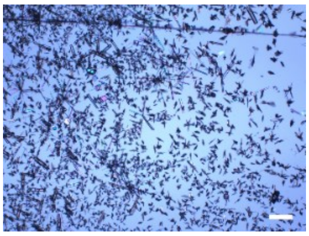	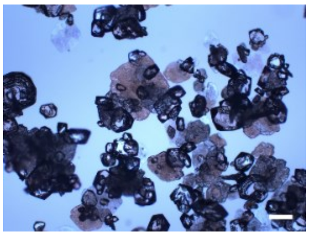
9	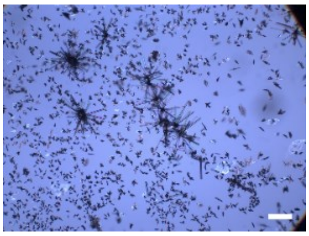	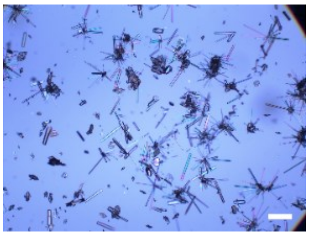	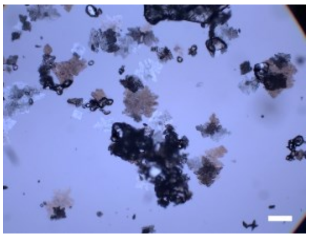

Figure S14. OM images of the PCA crystals produced by batch cooling crystallization with FUM using a 0.5 L-sized glass vessel in Expt. 4 to 9 (scale bar = 200 μm).

Expt.	At induction temp.	5 min after induction	At the end
10	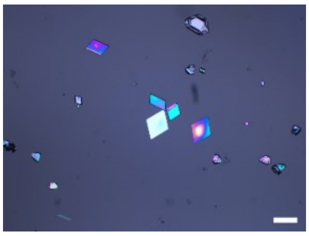	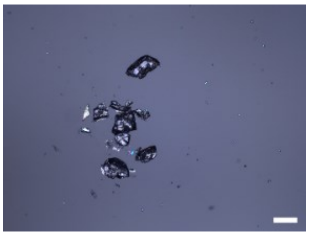	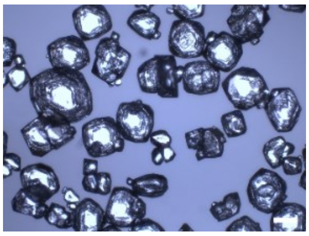
11	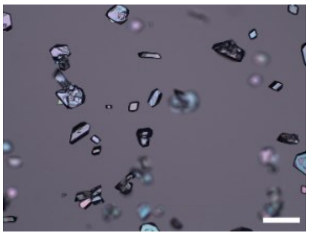	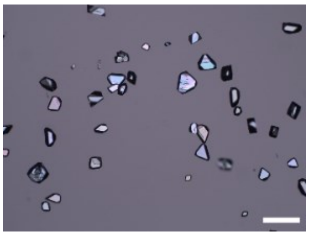	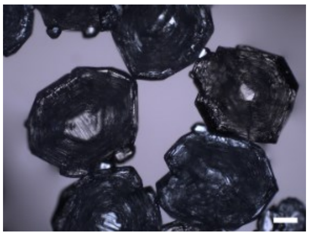
12	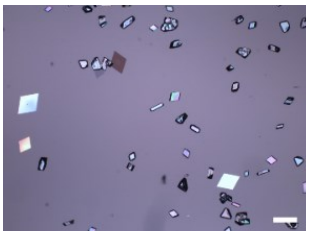	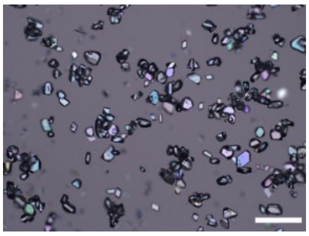	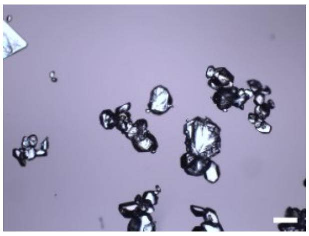
13	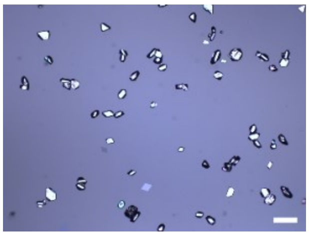	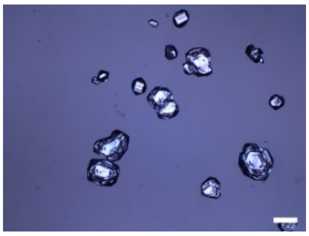	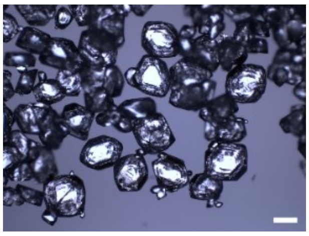
14	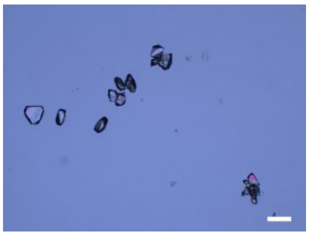	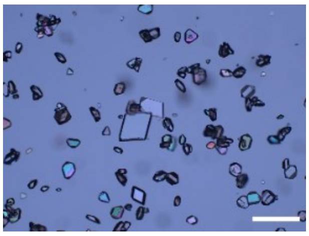	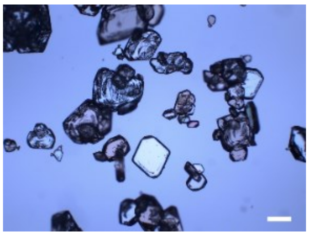
15	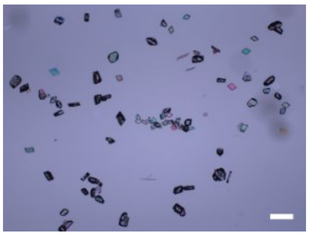	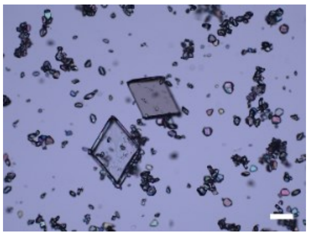	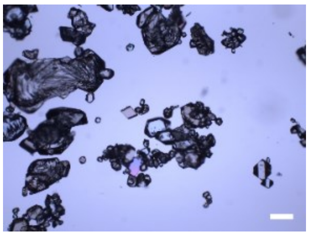

Figure S15. OM images of the PCA crystals produced by batch cooling crystallization with OXA using a 0.5 L-sized glass vessel in Expt. 10 to 15 (scale bar = 200 μm).

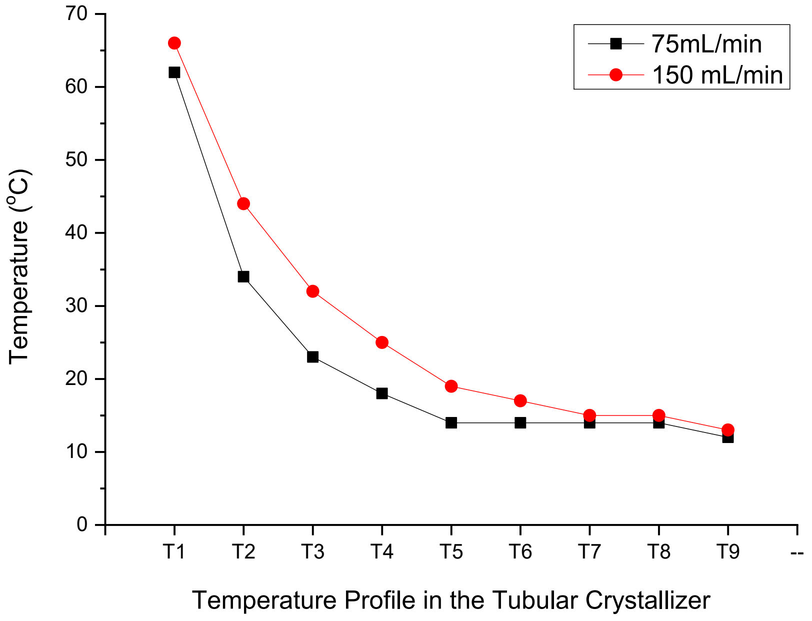

Figure S16. Temperature profiles determined at nine different positions in the tubular crystallizer with two flow rates of 75 and 150 mL/min.
